# Musical Museum: an integrative approach to emotional, intellectual and social stimulation for individuals with Alzheimer’s disease and related disorders and their caregivers

**DOI:** 10.3389/fneur.2026.1849901

**Published:** 2026-07-20

**Authors:** Jonathan Zhao, Melanie Zhang, Catherine Vidano, Clara Takarabe, Borna Bonakdarpour

**Affiliations:** 1 Mesulam Institute for Cognitive Neurology and Alzheimer's Disease, Northwestern University Feinberg School of Medicine, The Ken and Ruth Davee Department of Neurology, Chicago, IL, United States; 2 Northwestern University Feinberg School of Medicine, Chicago, IL, United States; 3 Northwestern University Medical Scientist Training Program, Chicago, IL, United States

**Keywords:** life enrichment, music medicine, music-based intervention, neurocognitive disorder, non-pharmacological intervention

## Abstract

**Introduction:**

Musical Museum is a concert series that features live music and short music appreciation lectures in a socially safe setting designed for individuals with neurocognitive disorders (NcDs) and their caregivers. This program was created to provide emotional well-being, intellectual stimulation and social interaction. In this manuscript, we present our viable methodology, satisfaction ratings, implementation practicality, exploratory outcomes, and plans for future quantitative research on this program.

**Methods:**

Participants, which included individuals with NcDs, their caregivers and other program supporters, experienced hourlong sessions with diverse music (both familiar and unfamiliar), enhanced by verbal introductions providing historical and musical context. Post-concert receptions encouraged social interaction. Anonymous surveys assessed satisfaction, pleasure, intellectual stimulation, likelihood to recommend and change in mood. Participants also provided open-ended feedback to evaluate logistics to determine practicality.

**Preliminary results:**

Musical Museum sessions require operational, logistical, and artistic components including venue setup, audiovisual support, communications, printed materials, staffing, and performance planning. Attendance data and expenses were tabulated to assess practicality and acceptability. In surveys, participants reported high satisfaction, pleasure, intellectual stimulation and likelihood to recommend. Participants’ perceived mood significantly improved post-session, and they showed appreciation of the social benefits of the program in open-ended questions.

**Discussion:**

Over 11 completed sessions, implementation of Musical Museum proved to be practical in terms of operational, logistical, and artistic components. Largely positive survey feedback and consistent interest and attendance suggested acceptability to individuals with NcDs and their caregivers. Future directions include a controlled quantitative trial integrating psychosocial and neurophysiologic measures to examine efficacy and underlying mechanisms beyond participant-reported acceptability as well as assessment of the program’s longitudinal impact on participant well-being.

## Background

As of 2026, more than 6–7 million Americans suffer from dementia, or neurocognitive disorder (NcD), which stems from a complex interplay of genetic and environmental factors, and growing evidence emphasizes the importance of modifiable lifestyle components in combating cognitive decline (1–3). A promising method to support cognitive well-being and delay decline is cognitive stimulation, defined as exposure to mentally challenging activities that maintain or improve functions such as memory, language, attention, and reasoning (4–6). Cognitive stimulation includes both the pleasure of grappling with new ideas and enhanced processing that occurs through discussion. These qualities are especially valuable for individuals with NcDs, for whom interventions must be mentally enriching and gratifying (7).

Lifelong learning programs are one method of cognitive stimulation. Pursued outside of formal schooling, lifelong learning has been associated with improved cognition, creativity, and social bonding (8). However, standard programs such as classes and lectures for older adults may prove inaccessible for individuals with cognitive impairment. At our institute, we observed that many patients opted out for these reasons, prompting us to create a program specifically tailored to their needs. In this manuscript, we discuss the methodology of such a program combining lifelong learning with music-based interventions, thus increasing accessibility and outcomes for the NcD population.

Music’s therapeutic potential for emotional regulation in individuals with NcDs is well-documented (9). One study provided biweekly music therapy to 104 elderly patients with NcDs and observed improved mood and short-term recall, particularly in those with mild to moderate impairment (10). Another found that active singing and passive listening independently reduced negative affect and supported emotional well-being (11). Programs such as the B Sharp Arts Engagement program offer further promise: participants attending Fort Collins Symphony concerts in Colorado over 10 months showed cognitive improvement (12). Music can also uniquely evoke autobiographical memories, which is crucial when voluntary recall is difficult for persons with NcDs (13). Such events, however, may exceed the attention span or physical tolerance of individuals with NcDs and lack integrated support.

Recognizing these challenges, we created the “Musical Museum” program at Northwestern University to provide a live music experience integrating intellectual stimulation and social interaction for people with NcDs in the Chicago area. Led by the senior authors (BB, CT) and with support from the Mesulam Institute and premedical student volunteers, Musical Museum combines live music and interactive discussion into a NcD-friendly experience designed to stimulate emotionally and intellectually without overwhelming participants. Unlike other music-only programs, it includes real-time commentary on musical history and theory for intellectual enrichment.

This approach builds on earlier work such as Loewy et al. (14), whose program paired live performances with post-event workshops to encourage reflection and caregiver bonding. Because engagement occurred well after music exposure, however, real-time cognitive stimulation was limited. Musical Museum bridges this gap through simultaneous performance and guided interaction.

Another component of the Musical Museum program is dedicated time and space for participants to connect with program staff, musicians, and each other. Compared to recorded music therapy, live music events may be particularly enriching for individuals with NcDs by providing opportunities for social interaction (15, 16). Social interaction benefits individuals with NcDs by improving both cognitive performance and quality of life, and interactions outside of the primary caregiving context (e.g., the home) may be especially rewarding (17). By integrating post-performance Q&A sessions and receptions, Musical Museum provides opportunities for social engagement that cannot be found in programs focused on recorded music or classroom instruction.

Equally important is our emphasis on NcD-friendly environments, which is guided by the WHO’s Age-Friendly Cities framework (18). These programs recognize the diverse manifestations of NcDs and provide welcoming infrastructure, such as clear signage, trained staff, and accessibility features, to support autonomy and dignity for these individuals.

In this manuscript, Musical Museum is presented as an emotionally and intellectually engaging program that integrates music, education, and dialogue for people with NcDs in a socially safe environment. We offer a detailed methodology of Musical Museum and its logistics as well as preliminary survey data to demonstrate acceptability by the audience. We will also discuss future goals for this project.

## Methods

### Participants

Individuals with NcDs and their caregivers learned about Musical Museum from Northwestern Medicine Neurobehavior and Memory Clinic clinicians. In their recommendations for healthy living for NcDs, Musical Museum was given as one of many options for maintaining cognitive well-being. The Mesulam Institute for Cognitive Neurology and Alzheimer’s disease maintains a mailing list, and those on the mailing list also received invitations. The mailing list includes individuals and organizations such as senior’s centers, community partners, and memory facilities across the Chicago metropolitan area. Musical Museum was advertised at internal events such as the Mesulam Institute’s Annual Alzheimer Day Conference, which is attended by clinicians, individuals with NcDs and their caregivers, as well as individuals working in NcD-adjacent fields (e.g., memory care facilities, retirement homes). Participants were accompanied by caregivers, family members and/or friends. We obtained permission from Northwestern Medicine to launch the Musical Museum as a disability-informed supportive program.

### Musical museum setup

Other than the first session, which was held over Zoom due to the COVID-19 pandemic, all sessions were in person. Venues for the first ten sessions were auditoriums on the Northwestern Feinberg School of Medicine campus. As it became more popular, the program was expanded to community and cultural venues that were disability friendly as well as geographically accessible. Audience members registered by responding to the email invitations. Emails were sent to both individuals with NcDs and their caregivers. All events began at 2:00 p.m. to avoid potential sundowning behavior among participants with NcDs; the formal program duration was 45 min with approximately 10–15 min of verbal engagement between the audience and artists via a question-and-answer period. Immediately after the question-and-answer period was the catered reception in which participants socialized on average for 30 min to an hour.

At the event, attendees checked in upon arrival. The registration table had two team members present. Individuals with NcDs often experience spatial disorientation and wayfinding impairment in new environments, so 2–4 staff members were available to orient and support the audience. Each performance was recorded and edited for posting on our YouTube channel (@NorthwesternMusicMedicine). Accessible seating was reserved for individuals with disabilities. By extending access to supportive social environments, the program addresses critical psychosocial dimensions often eroded by NcDs. Paper programs (including lyrics when applicable) were provided to all participants along with post-program surveys.

### Music selection

Each Musical Museum session is built around a theme. Program themes integrated philosophical concepts such as love, loss, and time, with culturally significant periods for older adults, notably the 1960s and 1970s. The themes and instrumentations for all sessions can be found in Table 1. Selections were intentionally brief and generally under 3 minutes to maintain engagement given the potential impact of NcDs on attention. Most programs incorporated a mix of familiar and unfamiliar music. Familiar music was defined as music that would have been popular during the audience’s early to late adolescence, and this period is often called the “reminiscence bump.” Previous research shows that familiar music may provide the benefits of not only improving autobiographical memory but also improving self-consciousness and reducing agitation (19–21). The music was tailored to our audience (individuals with NcDs and caregivers) based on our previous standardized surveys with the Northwestern Medicine patient population (22). During that study, patients were interviewed and filled out a music preference questionnaire that helped us identify appropriate songs and pieces. Unfamiliar music was generally tonal and possessed melodies that were culturally diverse and at times based on traditional folk tunes from a variety of geographical regions. When necessary, pieces were arranged, and composers and/or arrangers were sometimes involved in the process.

**Table 1:** Theme, music genre and instrumentation of each Musical Museum session.

#	Name	Description	Instrumentation
1	A journey	Classical and American contemporary	Viola, piano
2	Deep calm	Slow classical and contemporary	Viola, cello, piano
3	French baroque art song	French baroque	Voice, lute, guitar, theorbo
4	Viennese masters	Classical by Mozart and Schubert	violin, piano
5	Southside jazz	Jazz	Voice, guitar, bass guitar, drums, keyboard
6	Many faces of love	Classical and American contemporary	Voice, piano
7	Visit to the 1960s and 70s	American contemporary from the 1960s and 70s	Viola, synthesizer
8	Crossover music from around the world	Contemporary classical and Japanese video game music	Flute, viola, piano
9	Selections from songbook bossa nova	Brazilian bossa nova	Voice, guitar
10	Operatic celebration: Italian and polish classics	European opera	Voice, piano
11	Sweet home Chicago jazz	Jazz by Chicago-based artists	Voice, guitar, bass guitar, drums, keyboard

The structure of the program was important; the initial and final selections were deeply familiar and nostalgic, while the musical selections in the middle of the program were sometimes unfamiliar and more challenging in terms of rhythmic complexity while always diatonic. Diatonic music refers to the system of tonal organization used in most Western classical, folk and popular music. It is based on seven-note scales like the major and minor scales, built from specific patterns of whole and half steps without incorporating chromatic alterations beyond the key (23). This framework defines the harmonic and melodic characteristics of a piece and creates the familiar sense of key and harmony typical of Western tonal music. Extremely chromatic or atonal music was avoided.

Since Musical Museum also aims to reestablish a sense of belonging and comfort for individuals with NcDs and increase life space, each event began with an introduction outlining the NcD-informed framework to improve emotional well-being, provide cognitive stimulation, and foster social connection.

### Programming

Verbal introductions to musical selections highlighted historical, philosophical, and poetic aspects of the music. Spoken content was typically accompanied by visuals, including still images with minimal text, projected onto a large screen at the front of the room. The introductions were scripted, and the scripts were evaluated to ensure that audiences were intellectually stimulated but not overwhelmed. To simplify the scripts, complex grammatical structures such as highly embedded clauses were avoided, and words used in the script were mostly words with higher frequency of usage. Since auditory processing can be impaired in individuals with NcDs, presenters rehearsed scripted material to ensure controlled speech pacing and enriched prosodic variation with the aim of enhancing emotional resonance and affective engagement among audience members.

Programs were revised over time based on feedback from surveys. For example, verbal introductions were curtailed after some respondents reported that their length distracted from enjoyment of the music. Also, when possible, two to three songs were introduced together to avoid flow disruption. Participants indicated a desire for on-screen lyrics where applicable, which was implemented beginning with session 9. Until session 11, Musical Museum was held at facilities on Northwestern University’s campus in downtown Chicago. One respondent suggested that performances take place at other locations as well to promote accessibility for the wider community. For this reason, session 11 was held at a community center in the South Side of Chicago and turned out to be the most well-attended in-person performance up to that point.

### Opportunities for social engagement

Each Musical Museum offered two opportunities for social engagement. The first, shorter period is the question-and-answer session at the end of the performance where the presenting performer fields questions and comments from the audience. The second period of social engagement is the reception with catered food and drinks immediately afterward. This gives participants an opportunity to interact with performers, clinicians, and researchers hosting the event as well as other participants. Receptions averaged 1–2 h in length, with participants permitted to stay for as long as they wished.

### Surveys

Audience members, including individuals with NcDs and their caregivers, were given paper surveys to fill out at the end of the performances. Survey questions were refined over the course of 11 sessions. Surveys were self-completed by individuals with NcDs with help from their caregivers when needed. The language and structure of the surveys were designed with accessibility in mind (e.g., using short, grammatically simple sentences). Social desirability bias was mitigated by the anonymity of the surveys. The exact wording of each question and its corresponding answer choices can be found in Table 2. Of note, because the surveys were completed after the performances, questions 5 and 6 asked participants to rate their mood before and after each session retroactively.

**Table 2:** An example of language used in the survey instrument distributed at session 10.

#	Question	Answer choices
1	What was your overall satisfaction with the program?	1 Poor | 2 Fair | 3 Good | 4 Excellent | 5 Outstanding
2	To what degree did you find the music for this program pleasurable?	1 Poor | 2 Fair | 3 Good | 4 Excellent | 5 Outstanding
3	To what degree did you find the program intellectually stimulating?	1 Poor | 2 Fair | 3 Good | 4 Excellent | 5 Outstanding
4	How likely are you to recommend this program?	1 Very unlikely | 2 Unlikely | 3 Neutral | 4 Likely | 5 Very Likely
5	How would you rate your general feeling before the program?	1 Poor | 2 Fair | 3 Neutral | 4 Good | 5 Exceptional
6	How would you rate your general feeling after the program?	1 Poor | 2 Fair | 3 Neutral | 4 Good | 5 Exceptional
7	Please share any additional comments or suggestions.	(open ended)

This represents a complete list of all of the questions used to generate the data presented in this report.

Given that the survey questions changed over time, all of the questions that were used to collect the data presented in this report are as follows. Questions 1–6 utilized a balanced five-point Likert scale, with 1 being very negative and 5 being very positive. Questions 2, 5 and 6 evaluated perceived pleasure and mood to gauge emotional well-being, while question 3 evaluated perceived intellectual stimulation to gauge subjective cognitive effects. Question 7 was a space for optional, open-ended feedback. The full survey instrument used at session 10 is provided as Supplementary material S1 as an example. This version demonstrates the typical language and format that would have been experienced by respondents across all sessions.

Question 4 (rating likelihood to recommend) did not appear until session 3, and Questions 5 and 6 (rating mood before and after each session) did not appear until session 4 to include a pre−/post measure. Additionally, the first session survey contained a question regarding satisfaction with unfamiliar music selections as compared to familiar music, which was used to inform music selection in subsequent sessions.

The surveys were anonymous and therefore deidentified respondents, so this project does not meet the criteria for human subjects research. According to the approved Northwestern University IRB (IRB ID STU00203031), we were allowed to retrospectively assess results of surveys or clinical evaluations.

### Expenses and logistics

The Musical Museum program is largely funded by philanthropic support by individual corporate (e.g., Eisai Co., Ltd.) donors. The total cost per session ranged from approximately 1,800 to 2,800 USD. This wide range is primarily due to large variation in compensation for performers and ensemble size. The variable nature of this program suggests that it can be scaled according to the resources and abilities of the hosting institution or body as long as core components (live music, educational content and space for social interaction) are present.

An implementation checklist for hosting Musical Museum events can be found in Supplementary material S2. A general cost breakdown can be found in Supplementary material S3 and includes other expenses such as venue rental, audiovisual services, and catering, which were all provided through Northwestern University. Thus far, the program has been entirely staffed by 3–8 lab members and students.

### Practicality and cost-effectiveness

Practicality refers to the extent to which an intervention can be delivered as intended in real-world conditions using available personnel, infrastructure, time, and resources (24). Our goal was to evaluate whether Musical Museum could be implemented through consistent staffing, stable operations, and acceptable engagement and survey completion.

Program cost-effectiveness was evaluated by calculating the per-attendee cost across all 11 sessions at an average cost of $2,300 per session and a maximum venue capacity of 80 participants. Practicality thresholds were operationalized using benchmarks from the dementia program literature and our expectations with attendance at ≥60% of venue capacity considered acceptable (25). The event environment was designed to accommodate the sensory and socio-cognitive sensitivities associated with early to moderate-stage NcD; attendance density was intentionally maintained below maximum venue capacity levels to preserve spatial comfort and reduce the risk of overstimulation, anxiety, and environmental stress that may emerge in highly crowded settings for NcD populations. Per-attendee costs were benchmarked against published cost data for comparable dementia care modalities, including group music therapy (26), combined music therapy and cognitive stimulation (27), and structured caregiver support programs (28). A per-attendee cost of $50 was defined as acceptable and a cost of $41 as good (25).

### Statistical analysis

#### Likert variables

After combining individual responses across all sessions into one data set, medians and interquartile ranges (IQRs) were calculated for all Likert variables. DataTab was used to perform a Wilcoxon signed-rank test to compare results from questions 5 and 6 (see Table 2) (29).

The data from session 1, which was held on Zoom, were pooled with the data from all subsequent sessions, which were in person. In order to determine whether or not the delivery medium significantly altered participant outcomes, a sensitivity analysis was performed by recalculating descriptive statistics (median and IQR) for the entire data set excluding data from session 1. Questions 4, 5, and 6 (likelihood to recommend and pre/post mood) were excluded from this analysis because they did not appear on the survey for session 1.

#### Open-ended feedback section

A qualitative thematic coding analysis was conducted on open-ended participant comments following program attendance. Comments were coded inductively by authors CT and BB and then organized into program impact domains and engagement-related response themes. Inter-rater reliability was assessed using *Cohen’s κ,* calculated by comparing the proportion of observed agreement between the two observations to the agreement expected by chance: (*P_O_* − *P_e_*)/(1 − *P_e_*). Individual responses could contribute to multiple thematic categories. Feedback across all sections was also categorized into themes of benefitting emotional, intellectual, and social well-being specifically, while general positive comments were categorized as ‘general satisfaction.’ Suggestions were categorized as ‘advisory’ comments, which were then coded into four main subcategories including suggestions for improvement.

A word cloud was derived from the feedback using freewordcloudgenerator.com, an online tool, to find the words with the highest frequency of usage. Filler words were omitted for this purpose.

## Preliminary results

Survey respondents included dyads consisting of patients with NcDs and their caregiver as well as cognitively healthy program supporters. Surveys were completed individually unless a patient attended with a caregiver, in which case a single response was submitted by the dyad. Eighty seven percent (87%) of the survey responders were NcD-caregiver dyads. The mean age of the attendees was 72.5 (50–85) years. 62% of attendees were female, and 38% were male.

A total of 254 survey responses were collected from 551 attendances across the first 11 sessions with an average of 23 survey responses per session. The mean number of RSVPs per session was 66, while the mean number of attendees was 50, giving a 76% RSVP yield. Sessions were delivered consistently with stable performer recruitment, reliable venue access, and dependable caregiver transportation. Research staff maintained protocol fidelity with consistent music-introduction delivery.

From the total number of attendances, 187 (34%) were for 2 or more visits, and 139 (25%) were for 3 or more visits. Costs remained manageable at $37.70 per attendee. An attendance of 50 people per session was 62.5% of capacity (>60%), which was an acceptable turnout and ideal for fostering comfort for the individuals with NcDs by preventing overstimulation and agitation.

### Likert scale questions

**Figure 1:**
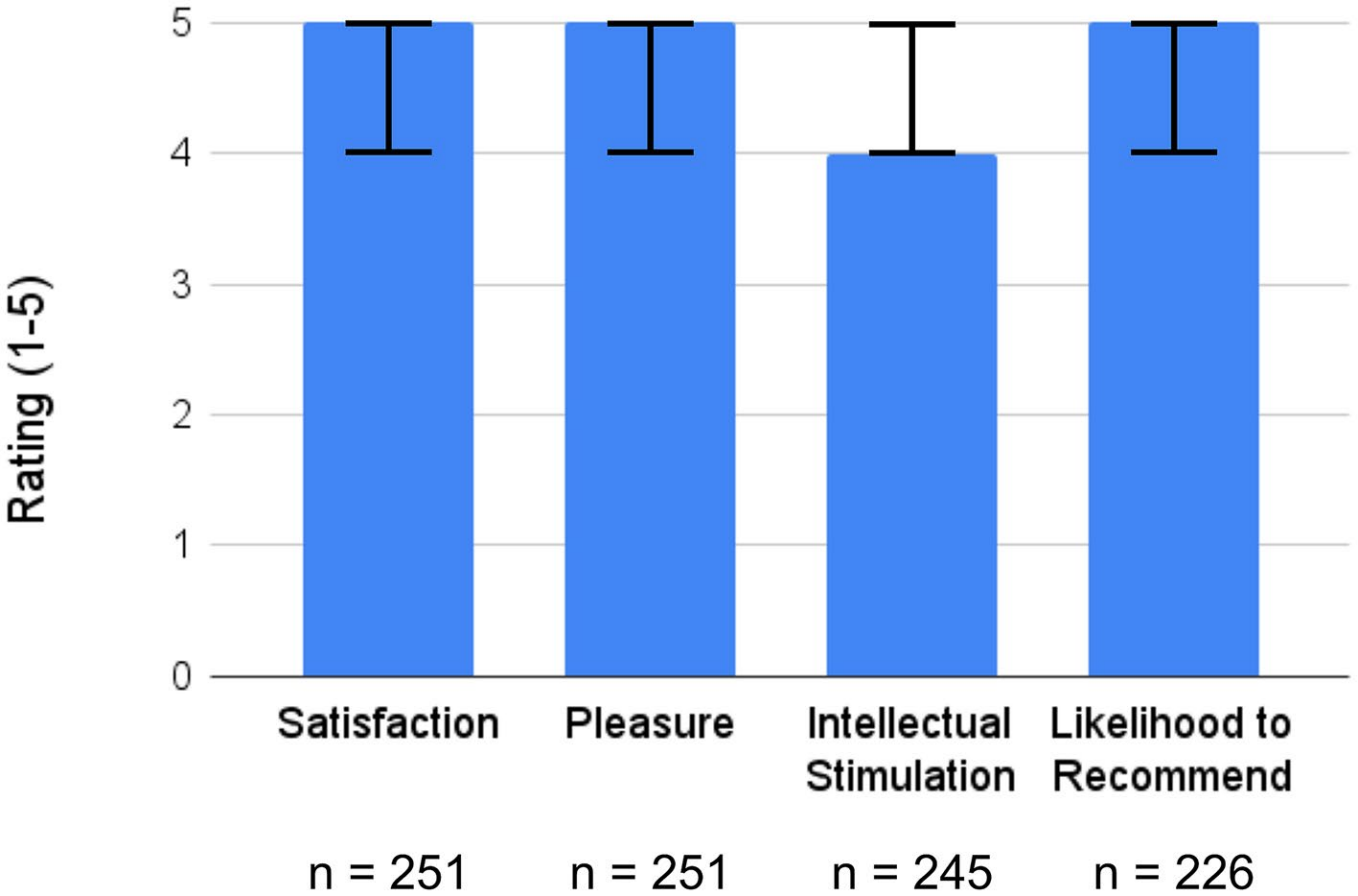
Survey results for the likert scale questions. Median self-reported measures are demonstrated together with interquartile range. The *n*-values in the figures differ from each other because question 4 on likelihood to recommend was not added until session 3, and questions 5 and 6 on pre/post mood were not added until session 4.

On a five-point Likert scale, median self-reported satisfaction, pleasure, intellectual stimulation and likelihood to recommend were 5 (IQR = 1), 5 (IQR = 1), 4 (IQR = 1), and 5 (IQR = 0) respectively (Figure 1). In session 1, a survey question asked respondents to rate their level of engagement on a scale from 1–5 with the unfamiliar music in the program, and the median rating was 4 (IQR = 1). Positive audience feedback on the unfamiliar selections of the first session prompted us to include unfamiliar music in all subsequent events.

A total of 254 survey responses were collected; surveys were completed anonymously, so it was not possible to determine whether some responses were contributed by the same individuals across multiple sessions. Therefore, potential effects of repeat respondents could not be assessed.

Attendance logs documented 551 total nonunique participants across sessions. This figure differs from the number of survey responses for two primary reasons. First, dyads were recorded as separate individuals for attendance purposes. Second, not all participants or dyads completed and returned surveys.

The n-values reported in Figure 1 also differ both from the total number of survey responses and from one another. This variation reflects two factors. First, certain survey items were introduced after the study had begun: Question 4 (likelihood to recommend) was added in Session 3, whereas Questions 5 and 6 (pre/post mood ratings) were added in Session 4. These staggered additions account for the majority of discrepancies in n-values across analyses. Second, some returned surveys were incomplete, with respondents omitting individual items for unknown reasons. Accordingly, the n-values reported for each figure represent the total number of valid responses obtained for the corresponding survey items.

Sensitivity analysis revealed that the inclusion of data from session 1 (Zoom instead of in person) did not affect overall ratings. Medians and IQRs for satisfaction, pleasure, and intellectual stimulation were the same for the full dataset (*n* = 11) compared to the in-person-only subset (*n* = 10).

**Figure 2:**
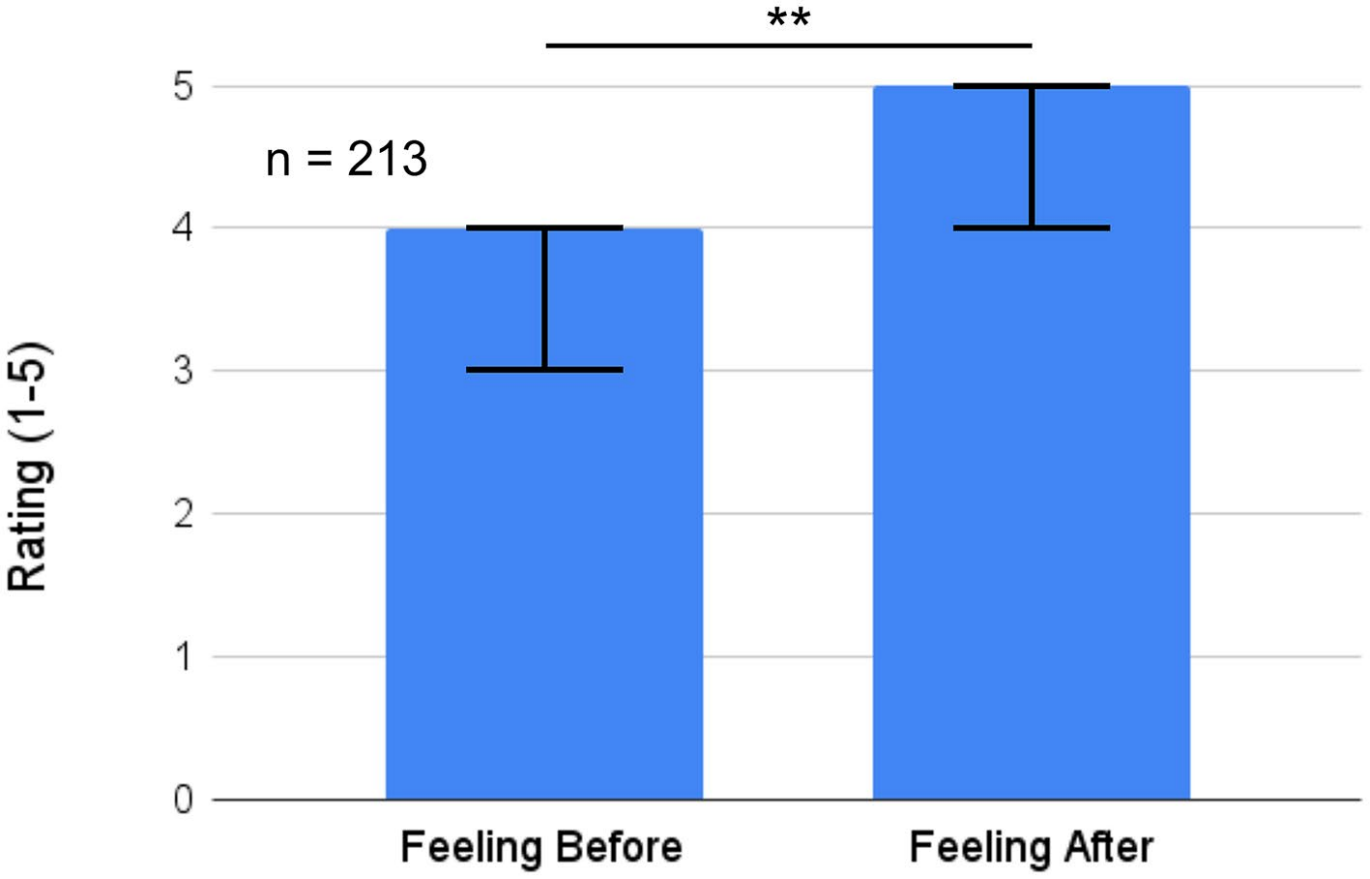
Ratings of “General Feeling” before and after the musical museum program on a five-point Likert scale, where 1-Poor; 2-Fair; 3-Neutral; 4-Good, 5-Exceptional. There was an improvement in “General Feeling” as reported by the audience that was shown to be statistically significant using a Wilcoxon signed-rank test.

The median retrospective self-reported pre-session mood on a five-point Likert scale was 4 (IQR = 1), while the median post-session mood was 5 (IQR = 1). The average post-session mood was significantly higher than the pre-session mood, as shown through a Wilcoxon signed-rank test (*p* < 0.001; W = 52) (Figure 2). The effect size was large and positive (*r* = 0.77), indicating a consistent subjective improvement in mood following the sessions.

### Open-ended questions

**Figure 3:**
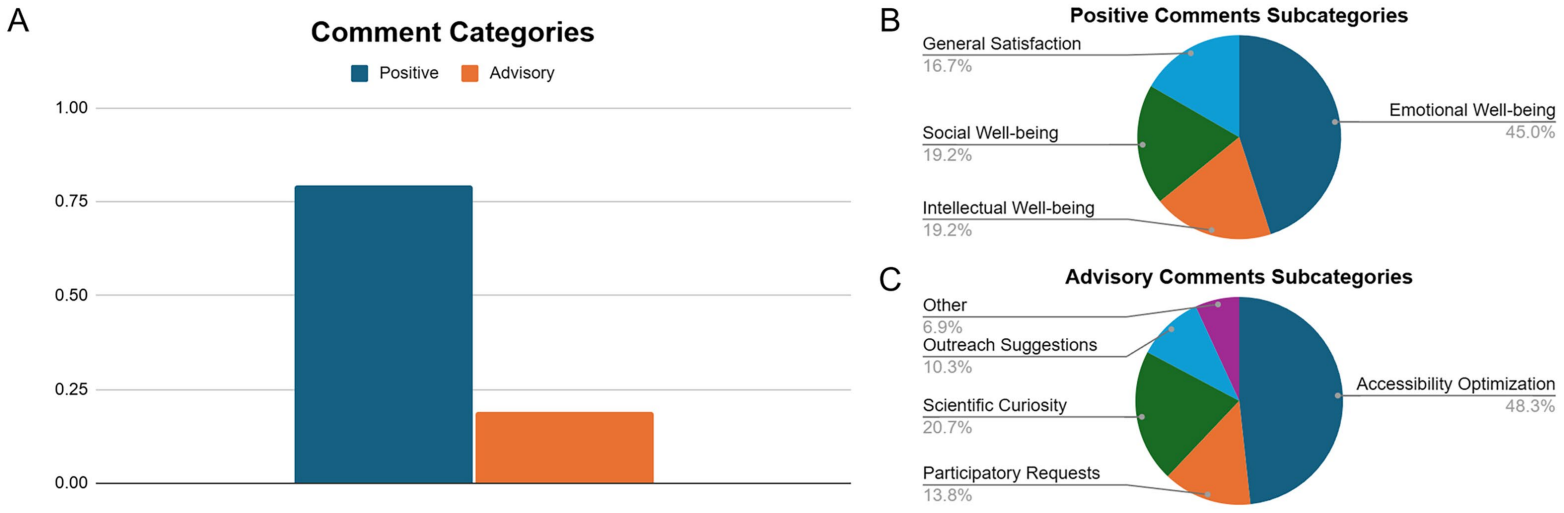
Distribution of free responses to open-ended questions. (A) Distribution of positive and advisory comments. **(B)** Subcategories of positive comments, which include references to emotional, social, and intellectual well-being; also included are comments on general satisfaction. **(C)** Breakdown of advisory comments, which include references to accessibility optimization, scientific curiosity, participatory requests, outreach suggestions, and “other”.

Thematic analysis of open-ended responses indicated that 120 (79.0%) comments were positive and 29 were advisory (19.0%), with Cohen’s *κ* of 0.933 (Figure 3A). 45.0% of *positive comments* were related to emotional well-being as a central benefit of the program, 19.2% to social connection, 19.2% to intellectual engagement, and 16.7% reporting general satisfaction (Cohen’s *κ =* 0.937*)* (Figure 3B).

*General positive comments:* The rest of the positive responses were comments pointing to the audience’s general satisfaction (e.g., “Great program,” “This was wonderful”) rather than emotional well-being, as they did not explicitly describe participant experience or specific emotional effects. This approach avoided overestimating emotional impact based on nonspecific expressions of approval.

Emotional well-being comments reflected positive affective shifts, including relaxation, enjoyment, and emotional release, indicating perceived mood regulation associated with program participation. Intellectual well-being comments reflected cognitive engagement with the program’s interpretive and educational dimensions, including historical context, reflections on the meaning of compositions, connections to broader world events, and thematic integration across the program. Social well-being comments reflected the value of shared experience and interpersonal connection, with participants noting enjoyment and opportunities to interact with others affected by NcDs.

*Advisory comments* largely reflected engagement rather than dissatisfaction. They were focused on accessibility optimization (48.3%), expressions of curiosity regarding the neuroscientific basis of the intervention (20.7%), interest in more participation in music-making (13.8%), and outreach suggestions to broaden inclusion efforts (10.3%) (Cohen’s *κ =* 0.947; Figure 3C).

“Accessibility optimization” refers to participant suggestions aimed at improving cognitive, auditory, or perceptual processing ease of the program, particularly for individuals with NcDs and age-related sensory changes. These comments suggested adjustments to pacing, volume, visual supports (e.g., projected lyrics), sequencing of explanations, and communication clarity, and did not refer to physical disability accommodations.

**Figure 4:**
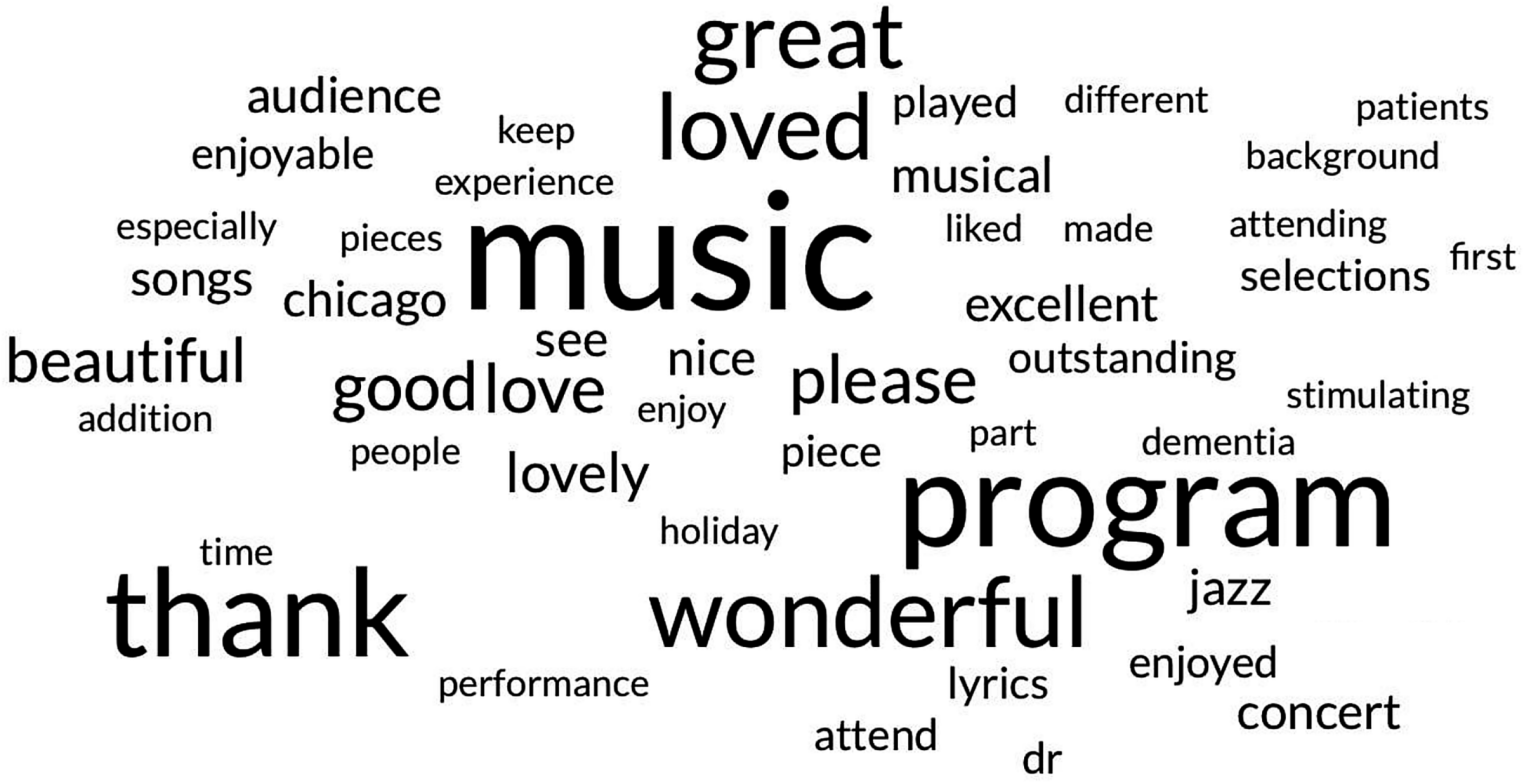
Word cloud showing frequency of keywords in open-ended feedback across all sessions. Larger font indicates higher frequency of use.

Figure 4 demonstrates the words used with the highest frequency based on the word cloud (e.g., “great,” “thank,” “loved,” “wonderful,” “beautiful”). Table 3 displays the open-ended feedback categorized into five themes.

**Table 3:** Examples of open-ended responses by category.

Category	Example phrases
Emotional well-being	“This program provided peace, relaxation, and occasionally a cathartic release of tears.”
Intellectual well-being	“I especially loved the historical context about the pieces It made me appreciate the new pieces more and understand the ones I already knew more.”
Social well-being	“Wonderful opportunity to expand our contacts with individuals undergoing similar experiences.”
General satisfaction	“Great program! Thank you!”
Accessibility optimization	“Speak much slower, and play much slower.”
Scientific curiosity	“I’d love to know more about the scientific linkage between the brain and this particular program.”
Participation requests	“I would have liked to add my voice. I sing well.”
Outreach suggestions	“More outreach to diverse, e.g., African-American elders please!”

## Discussion

This manuscript outlines a comprehensive methodological framework for Musical Museum, a socio-musical intervention aimed at delivering integrative emotional, intellectual, and social stimulation for individuals with NcDs and their caregivers. The program was started and sustained due to a growing interest of individuals with NcDs and their caregivers who continually supported the series and expressed the desire for the program to continue. As the program expanded, external sponsors recognized its demonstrated success and subsequently provided financial support. Our survey results showed that most of the participants found the program pleasurable and intellectually stimulating, with high levels of satisfaction and likelihood to recommend according to our surveys. Participants reported significantly enhanced mood following each event. Open-ended feedback was overwhelmingly positive, with a focus on enhanced emotional, intellectual, and social well-being. Some respondents also offered suggestions for improvement, which we addressed to the best of our ability.

### Practicality and cost effectiveness

The Musical Museum program demonstrated strong practicality across feasibility domains.

Across the first 11 thematically diverse concerts, a mean RSVP-to-attendance of 76% represents a high level of participation for a dementia population, in which attendance is frequently affected by health fluctuations, caregiver availability, transportation difficulties, sudden illness, weather conditions, and other logistical barriers. An attendance of >60% capacity was determined to be acceptable per our threshold.

Sessions were delivered consistently with stable performer recruitment, reliable venue access, and dependable caregiver transportation. Research staff maintained protocol fidelity, enabling reproducible session structure and consistent music-introduction delivery. Recruitment pipelines supported sustained engagement, reflected in a 34% retention rate for two or more sessions (187 participants), with additional retention at three or more sessions (25%), indicating meaningful multi-session participation given our restrictions. It is important to note that we have an opportunity to enhance program retention. At this stage, we did not have enough resources to maintain more regular sessions (e.g., weekly). With bimonthly sessions, participants were not required to attend repeated sessions as part of a treatment regimen. In addition, the theme of the program changed between sessions to offer variety. Participants of one session may have not been interested in the themes of a subsequent session, which may have impacted attendance. Attendance fluctuations were also partially attributable to seasonal migration patterns among older adult participants who temporarily relocate to warmer areas during winter months.

Costs remained manageable at $37.70 per attendee, which was acceptable and competitive to other programs (individual music therapy sessions typically run $80–$150 per session, adult day program activities average $50–$100 per session, and group dementia recreation programs benchmark at $50–$75 per person). Importantly, our events were offered at no cost to the participants to promote accessibility (26–28). Survey completion of 254, given dyadic responses, is considered acceptable. Overall, the program met published practicality benchmarks.

Although there have been a few pilot studies investigating community-based music programs in individuals with NcDs, Musical Museum was unique in featuring live music programming and interactive discussions specifically curated and formatted with clinical consideration for those with NcDs and their caregivers (12, 14, 30). The combination of familiar and unfamiliar music was well received by the audience. Preserved responsiveness of individuals with NcDs to familiar music, which represents a “reminiscence bump,” is well known in the literature, so familiar music is an excellent method to engage individuals with NcDs who are more advanced (13, 31, 32). However, most individuals with mild to moderate NcDs can still enjoy unfamiliar melodic pieces (33). Using unfamiliar music provides representation from different cultures and opportunities for lifelong learning and cognitive stimulation if pieces are arranged in the usual Western style.

It should also be noted that while most Musical Museum sessions utilized Northwestern University’s infrastructure and resources, institutional resources are not necessary to reproduce this program’s core features. Community spaces, such as the one that hosted session 11, are acceptable alternatives to the academic auditoriums we utilized for our other performances as long as they have sufficient occupancy and audiovisual equipment. Our catering was a paid University service, but light refreshments may also be personally provided by event hosts to reduce monetary and labor costs. Supplementary material S2, S3 contain a complete list of requirements for recreating Musical Museum or a similar program based on our experience. The scale of the program may be adjusted depending on the ability of the host, provided that key components such as live music, educational content, and opportunities for social interaction are preserved.

### Likert scale measures

All four Likert scale measures (satisfaction, pleasure, intellectual stimulation, and likelihood to recommend) were rated highly according to response medians (≥4) (Figure 1). These data suggest that participants enjoyed not only the musical performances but also the verbal introductions for their educational value.

Findings of high satisfaction and pleasure when listening to the performances are consistent with previous research showing that both individuals with NcDs and their caregivers derive enjoyment and happiness from listening to music (34). Similarly, retrospective pre−/post-evaluation showed a statistically significant improvement in participants’ general feeling ratings, which is consistent with studies demonstrating that receptive live music interventions can improve mood and elevate emotions (Figure 2) (10, 11).

The inclusion of verbal introductions to each piece, which represented the intellectual stimulation component, was a unique feature of Musical Museum series. Introductions were facilitated by visual slides and captions for individuals with aphasia or impairment in their auditory working memory. While there have been studies of music interventions in group settings for individuals with NcDs in the form of group singing, personalized music listening, and playlists, none used verbal discussions of music or assistive visuals (35–37). Based on our data showing high levels of self-reported intellectual stimulation overall, these features can be added to concert-based programs for individuals with NcDs as a form of lifelong learning and cognitive stimulation for both them and their caregivers.

The median rating of intellectual stimulation was lower than other measures. This could be reflective of the challenges individuals with NcD experience. Alternatively, the information load or presentation style may need to be adjusted (38). We plan to further investigate this area to improve the audience experience of intellectual stimulation.

### Open-ended responses

General positive feedback as well as specific mentions of emotional and intellectual well-being on the surveys is largely consistent with our findings from Likert scale measures on satisfaction, pleasure, and intellectual stimulation (Figure 1). These findings are also consistent with our previous study of the effect of group singing in individuals with NcDs and older adults (39). Music has a wide effect on many cognitive domains; however, its effect on emotions is the most significant (40). Through auditory frontal pathways, music also taps into further cognitive processing in the brain, a feature that was boosted by introductory remarks and hence assisted the audience by increasing their attention to the music’s technical aspects and historical background (41).

Participants described the music program as a powerful source of social well-being, emphasizing the joy of shared musical experiences, the comfort of being physically present with others, and the strong sense of community that emerged as audiences grew and returned each month. The dementia-informed structure of the program helped individuals feel successful and confident in social settings, while anticipation of future sessions provided meaningful “future thinking.” Attendees highlighted the pleasure of connecting with others who share similar experiences, noting that the concerts created a welcoming environment for people with dementia and their care partners. These observations align with research showing that shared music experience enhances social connectedness and emotional well-being in dementia (42), supports identity and communication (43), improves quality of life (44), and strengthens relationships within dementia communities (45).

Accessibility optimization referred to participant suggestions aimed at improving cognitive, auditory, or perceptual processing ease of the program, particularly for individuals with neurocognitive disorders and age-related sensory changes. These comments concerned adjustments to pacing, volume, visual supports (e.g., projected lyrics), sequencing of explanations, and communication clarity and did not refer to physical disability accommodations. With subsequent programs, we made every effort to respond to participant suggestions and requests. For example, where necessary, the pace of presentations was slowed, the material was repeated, or more information was communicated through simplified slides. The audiences’ expressions of scientific curiosity attests to their readiness to be involved in further collaborative scientific evaluations of Musical Museum, which will be invaluable as we prepare to define the mechanistic underpinning of the effect of such sessions using electroencephalography, measurement of heart rate variability, and blood pressure.

The audiences’ request to participate in music making was readily accommodated by the inclusion of songs for sing-along format with the audience. Outreach locations were broadened so that Musical Museums are now taking place not only in downtown Chicago but also in the South Side Bronzeville neighborhood, which is predominantly African American, and in the Bridgeport neighborhood, which is predominantly Chinese American. Musical Museum has also spread to Wisconsin (Brown and Door Counties), a state with a relatively high proportion of older adults. There has also been interest from geriatric and public health departments at other universities as well as symphonies that wish to develop similar programs at national and international locations, giving us an opportunity to increase our reach.

### Limitations

The goal of this manuscript was to introduce the methodology of the Musical Museum program, evidence for its practicality, and to provide some preliminary survey data. It is important to note that our findings are constrained by subjective surveys, lack of blinding, absence of control groups, and limited characterization of NcD subtypes. To move towards efficacious results across multiple sessions, we need to implement more frequent (e.g., weekly) sessions, which necessitates more financial support. In addition, objective physiologic measures, broader demographics, and scalable randomized models are needed to strengthen reliability and expand impact.

Utilization of a retrospective pretest of mood change rather than a true pretest is another limitation of this study. Because the surveys were to be completed at the end of each session, respondents were required to evaluate their pre-performance moods long after that time had passed, introducing the possibility of recall bias. In the future, we will request that respondents indicate their pre-session moods on the surveys before the performances.

While survey responses were completed by patient-caregiver dyads, we did not track how the dyads coordinated survey completion among themselves. In subsequent sessions, we will obtain NcD and caregiver responses separately. In cases where a caregiver assists the NcD participant with their responses, we will instruct the caregiver to prioritize the opinions of the individuals with NcDs. We will also include a box to check in the form indicating that the caregiver was assisting with survey completion.

From 551 total nonunique program attendees, we received only 254 completed surveys, giving an apparent response rate of approximately 46%. Two individuals (NcD and caregiver) frequently submitted one survey as a dyad, roughly halving the apparent number of respondents. While dissatisfaction could be one reason for not submitting survey responses, other reasons included leaving early to avoid rush hour traffic, insufficient number of blank surveys, participants attending without their reading glasses, or participants leaving for the reception, rather than being dissatisfied. Going forward, we will request separate responses from individuals with NcDs and their caregivers, and we will further encourage (within reason) the completion and returning of surveys so that a wide range of feedback is more accurately captured.

Another limitation of the current implementation is the absence of a standardized longitudinal treatment structure. Future studies should investigate the effects of a more formalized intervention model in which participants enroll in an 8–12 session course administered across approximately 2–3 months. Such an approach may permit improved participant retention, greater consistency of exposure, and more robust pre-post and longitudinal outcome analyses. Future scalability will additionally require dedicated administrative support for enrollment management, participant coordination, and operational oversight, as well as evaluation of sustainable funding or participant payment models.

### Conclusions and future directions

Musical Museum demonstrates that a live, curated music appreciation program can be implemented practically and is highly acceptable to individuals with neurocognitive disorders and their caregivers. Participants consistently reported mood improvement, intellectual engagement, and meaningful social interaction, illustrating the program’s value as an arts-based supportive intervention. Operational feasibility across multiple sessions further supports its sustainability. Building on these encouraging findings, future research will incorporate more frequent sessions toward achieving cumulative outcome measures, controlled designs, as well as psychosocial and neurophysiologic measures to evaluate efficacy, mechanisms of action, and long-term impact. These next steps will guide broader dissemination and inform the development of scalable, rigorous, and evidence-based music programs for NcD populations and their caregivers.

## Data Availability

The raw data supporting the conclusions of this article will be made available by the authors, without undue reservation.
