# Supplementary material for: Musical Museum: an integrative approach to emotional, intellectual and social stimulation for individuals with Alzheimer’s disease and related disorders and their caregivers
Source: Front Neurol. 2026 Jul 20;17:1849901. doi: 10.3389/fneur.2026.1849901 (PMC13429727; doi:10.3389/fneur.2026.1849901)
Supplement: Supplementary file 1 [file Data_Sheet_1.pdf]

Musical Museum 10 Survey

"Operatic Celebration"

1. **What was your overall satisfaction with the program?**

   1 Poor 2 Fair 3 Good 4 Excellent 5 Outstanding

2. **To what degree did you find the music for this program pleasurable?**

   1 Poor 2 Fair 3 Good 4 Excellent 5 Outstanding

3. **To what degree did you find the program intellectually stimulating?**

   1 Poor 2 Fair 3 Good 4 Excellent 5 Outstanding

4. **How likely are you to recommend this program?**

   1 Very unlikely 2 Unlikely 3 Neutral 4 Likely 5 Very Likely

5. **How would you rate your general feeling <u>before</u> the program?**

   1 Poor 2 Fair 3 Neutral 4 Good 5 Exceptional

6. **How would you rate your general feeling <u>after</u> the program?**

   1 Poor 2 Fair 3 Neutral 4 Good 5 Exceptional

7. **Please share any additional comments or suggestions:**

____

____

Supplement 1. Survey distributed to participants at session 10. *This session's survey contains every question used for data collection.*
