# Supplementary material for: Musical Museum: an integrative approach to emotional, intellectual and social stimulation for individuals with Alzheimer’s disease and related disorders and their caregivers
Source: Front Neurol. 2026 Jul 20;17:1849901. doi: 10.3389/fneur.2026.1849901 (PMC13429727; doi:10.3389/fneur.2026.1849901)
Supplement: Supplementary file 2 [file Data_Sheet_2.pdf]

1. Physical Space and Logistics

- Secure venue (auditorium or classroom) with adequate seating and accessibility.
- Confirm availability of audiovisual equipment (projector/screen for slides, live captioning capability).
- Arrange check-in station (table and signage).
- Coordinate catering services and refreshments for post-session reception.
- Schedule support services as needed (movers, housekeeping, audiovisual technicians).
- Ensure loading dock access for musical instruments and equipment.

2. Communications and Participant Materials

- Send confirmation and logistical details to musicians.
- Distribute reminder emails to registered participants.
- Prepare printed materials:
    - Audience handouts (programs with lyrics if applicable).
    - Surveys for post-session feedback.
- Provide clear signage and directions for attendees.

3. Performance and Session Documentation

- Develop and finalize presentation slides for music appreciation segments.
- Prepare a “Run of Show” document detailing timing and sequence of activities.
- Maintain RSVP list and attendance records for evaluation purposes.

Supplement 2. Implementation checklist for hosting Musical Museum sessions. *This checklist outlines essential logistical and communication steps for delivering Musical Museum sessions, ensuring reproducibility and fidelity to the intervention design.*
