# Supplementary material for: Musical Museum: an integrative approach to emotional, intellectual and social stimulation for individuals with Alzheimer’s disease and related disorders and their caregivers
Source: Front Neurol. 2026 Jul 20;17:1849901. doi: 10.3389/fneur.2026.1849901 (PMC13429727; doi:10.3389/fneur.2026.1849901)
Supplement: Supplementary file 3 [file Data_Sheet_3.pdf]

Resource and Cost Categories for Musical Museum Implementation (per session):		
<u>**Category**</u>	<u>**Description**</u>	<u>**Estimated cost**</u>
**Musician Fees**	Compensation for performers (including rehearsal time if applicable).	$500-$1500
**Venue Rental**	Auditorium or classroom reservation including movers, housekeeping, etc.	$500
**Audiovisual Services**	Technicians, equipment setup and live captioning (sometimes included in venue).	$200
**Catering**	Refreshments for post-session social reception.	$500
**Printing Services**	Programs, lyrics sheets, handouts and surveys.	$50
**Marketing / Communications**	Email campaigns, flyers and participant reminders.	Included in Alzheimer's Center budget
**Miscellaneous Supplies**	Check-in table materials: signage, name tags, pens, etc.	$50
**Labor**	Student assistants and lab volunteers supporting event setup and execution.	N/A
	**Total cost per session**	**$1,800-$2,800**

Supplement 3. Summary of the primary resource categories necessary for replicating Musical Museum sessions, including personnel, venue, audiovisual support and material costs. *Estimated costs should be adapted based on local pricing and institutional resources.*
